# 4,8-Dimeth­oxy­furo[2,3-*b*]quinoline (γ-fagarine)

**DOI:** 10.1107/S1600536811025062

**Published:** 2011-07-06

**Authors:** Yong Liu, Kou Wei, Jianshe Yang

**Affiliations:** a Medical College, Northwest University for Nationalities, Lanzhou 730030, Gansu Province, People’s Republic of China; b Life Sciences College, Northwest Normal University, Lanzhou 730030, Gansu Province, People’s Republic of China

## Abstract

The title mol­ecule, C_13_H_11_NO_3_, a natural compound extracted from *Phellodendron chinense*, exhibits a near planar framework: the mean deviations from the furo[2,3-*b*]quinoline ring system and from the whole mol­ecule (not including the H atoms) are 0.006 and 0.062 Å, respectively.

## Related literature

For the anti-HIV properties of furoquinolines, see: Wang *et al.* (2009 ▶); Cheng *et al.* (2005 ▶). For a related furoquinoline structure, see: Napolitano *et al.* (2003 ▶).
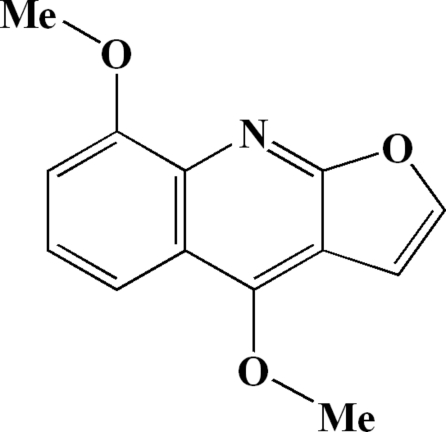


## Experimental

### 

#### Crystal data

C_13_H_11_NO_3_
*M* *_r_* = 229.23
Orthorhombic, 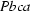

*a* = 12.491 (5) Å
*b* = 12.155 (5) Å
*c* = 14.466 (5) Å
*V* = 2196.4 (14) Å^3^
*Z* = 8
Mo *K*α radiation
μ = 0.10 mm^−1^
*T* = 296 K
0.25 × 0.22 × 0.21 mm

#### Data collection

Bruker APEXII CCD diffractometer
Absorption correction: multi-scan (*SADABS*; Sheldrick, 1998 ▶) *T*_min_ = 0.976, *T*_max_ = 0.979
11659 measured reflections
2047 independent reflections
1278 reflections with *I* > 2σ(*I*)
*R*_int_ = 0.068

#### Refinement

*R*[*F* ^2^ > 2σ(*F* ^2^)] = 0.044
*wR*(*F* ^2^) = 0.129
*S* = 1.05
2047 reflections
157 parameters
H-atom parameters constrained
Δρ_max_ = 0.14 e Å^−3^
Δρ_min_ = −0.12 e Å^−3^

### 

Data collection: *APEX2* (Bruker, 2005 ▶); cell refinement: *SAINT* (Bruker, 2001 ▶); data reduction: *SAINT*; program(s) used to solve structure: *SHELXS97* (Sheldrick, 2008 ▶); program(s) used to refine structure: *SHELXL97* (Sheldrick, 2008 ▶); molecular graphics: *SHELXTL* (Sheldrick, 2008 ▶); software used to prepare material for publication: *SHELXTL*.

## Supplementary Material

Crystal structure: contains datablock(s) global, I. DOI: 10.1107/S1600536811025062/bh2360sup1.cif

Structure factors: contains datablock(s) I. DOI: 10.1107/S1600536811025062/bh2360Isup2.hkl

Supplementary material file. DOI: 10.1107/S1600536811025062/bh2360Isup3.cml

Additional supplementary materials:  crystallographic information; 3D view; checkCIF report

## Figures and Tables

**Fig. 1.**
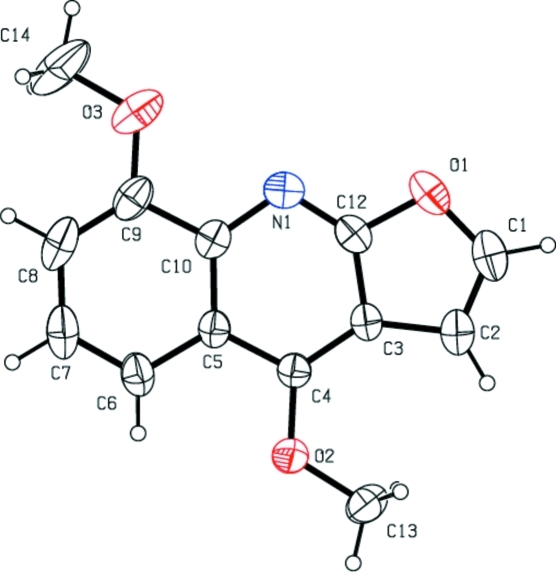
The molecular structure of I with, displacement ellipsoids drawn at the 30% probability level. H atoms are presented as small spheres of arbitrary radius.

## supplementary crystallographic information

### Comment

Furoquinoline is a planar unit, and its derivatives have been found to be potent anti-HIV compounds (Wang *et al.*, 2009; Cheng *et al.*, 2005). In the course of exploring new anti-HIV agents, we obtained a natural product, 4,8-dimethoxyfuro[2,3-*b*]quinoline, from *phellodendron chinense*. Here we report the structure and isolation of title compound.

The furo[2,3-*b*]quinoline ring system is near planar, exhibiting mean deviation of 0.006 Å. The two methoxy substitutional groups are nearly coplanar with the furo[2,3-*b*]quinoline ring system. The maximum distance from the four atoms of the two methoxy groups to the furo[2,3-*b*]quinoline framework mean plane is 0.300 (6) Å, for atom C14.

The title molecule crystallizes in space group *Pbca*, which is different from that of the closely related 4,7,8-trimethoxyfuro[2,3-*b*]quinoline (*P*2_1_/*c*, Napolitano *et al.*, 2003). There are no classic hydrogen bonds in the crystal structure of the title compound.

### Experimental

*Phellodendron chinense* (500 g) and 85% ethanol (1 L) were added to a 2 L flask. After refluxing the mixture for 5 h, the mixture was cooled to 300 K and filtrated. After the filtrate being condensed to 100 mL in water bath, the remains were extracted with ethyl acetate and dried over Na_2_SO_4_. After removing the solvent, the crude product was purified by a silica gel column using hexane/acetone, 3/1, as eluent, to give the title compound (1.10 g). Then the compound was dissolved in THF, and colorless crystals were formed on slow evaporation, at room temperature over one week.

### Refinement

All H atoms were placed in geometrically calculated positions and refined using a riding model with C—H = 0.93 (for aromatic H) or 0.96 Å (for methyl groups), with *U*_iso_(H) = 1.2*U*_eq_(C) or *U*_iso_(H) = 1.5*U*_eq_(C of methyl).

### Crystal data

**Table:**

C_13_H_11_NO_3_	*F*(000) = 960
*M**_r_* = 229.23	*D*_x_ = 1.386 Mg m^−^^3^
Orthorhombic, *P**b**c**a*	Mo *K*α radiation, λ = 0.71073 Å
Hall symbol: -P 2ac 2ab	Cell parameters from 1884 reflections
*a* = 12.491 (5) Å	θ = 2.7–24.2°
*b* = 12.155 (5) Å	µ = 0.10 mm^−^^1^
*c* = 14.466 (5) Å	*T* = 296 K
*V* = 2196.4 (14) Å^3^	Block, colourless
*Z* = 8	0.25 × 0.22 × 0.21 mm

### Data collection

**Table:**

Bruker APEXII CCD diffractometer	2047 independent reflections
Radiation source: fine-focus sealed tube	1278 reflections with *I* > 2σ(*I*)
graphite	*R*_int_ = 0.068
φ and ω scans	θ_max_ = 25.5°, θ_min_ = 2.7°
Absorption correction: multi-scan (*SADABS*; Sheldrick, 1998)	*h* = −15→13
*T*_min_ = 0.976, *T*_max_ = 0.979	*k* = −13→14
11659 measured reflections	*l* = −16→17

### Refinement

**Table:**

Refinement on *F*^2^	Secondary atom site location: difference Fourier map
Least-squares matrix: full	Hydrogen site location: inferred from neighbouring sites
*R*[*F*^2^ > 2σ(*F*^2^)] = 0.044	H-atom parameters constrained
*wR*(*F*^2^) = 0.129	*w* = 1/[σ^2^(*F*_o_^2^) + (0.0376*P*)^2^ + 0.5982*P*] where *P* = (*F*_o_^2^ + 2*F*_c_^2^)/3
*S* = 1.05	(Δ/σ)_max_ = 0.001
2047 reflections	Δρ_max_ = 0.14 e Å^−^^3^
157 parameters	Δρ_min_ = −0.12 e Å^−^^3^
0 restraints	Extinction correction: *SHELXL97* (Sheldrick, 2008), Fc^*^=kFc[1+0.001xFc^2^λ^3^/sin(2θ)]^-1/4^
0 constraints	Extinction coefficient: 0.0037 (8)
Primary atom site location: structure-invariant direct methods	

### Fractional atomic coordinates and isotropic or equivalent isotropic displacement parameters (Å^2^)

**Table:**

	*x*	*y*	*z*	*U*_iso_*/*U*_eq_	
C1	1.0282 (3)	0.2044 (2)	−0.15171 (19)	0.0791 (8)	
H1	1.0559	0.2307	−0.2072	0.095*	
C2	1.0798 (2)	0.21016 (18)	−0.07253 (17)	0.0654 (7)	
H2	1.1474	0.2401	−0.0627	0.078*	
C3	1.01087 (17)	0.16075 (15)	−0.00386 (14)	0.0474 (5)	
C4	1.00926 (16)	0.13961 (15)	0.08969 (14)	0.0476 (5)	
C5	0.91729 (17)	0.08795 (16)	0.12813 (14)	0.0484 (5)	
C6	0.9100 (2)	0.06319 (18)	0.22317 (16)	0.0651 (7)	
H6	0.9662	0.0807	0.2627	0.078*	
C7	0.8204 (2)	0.0136 (2)	0.2568 (2)	0.0819 (9)	
H7	0.8159	−0.0021	0.3196	0.098*	
C8	0.7351 (2)	−0.0142 (2)	0.1989 (2)	0.0835 (9)	
H8	0.6746	−0.0480	0.2233	0.100*	
C9	0.7403 (2)	0.00804 (18)	0.1070 (2)	0.0684 (7)	
C10	0.83121 (17)	0.06121 (16)	0.06827 (16)	0.0524 (6)	
C12	0.91869 (19)	0.12867 (17)	−0.05247 (15)	0.0544 (6)	
C13	1.18328 (18)	0.2155 (2)	0.12003 (19)	0.0788 (8)	
H13A	1.2177	0.1688	0.0755	0.118*	
H13B	1.2303	0.2269	0.1716	0.118*	
H13C	1.1667	0.2850	0.0920	0.118*	
C14	0.5786 (3)	−0.0878 (3)	0.0754 (3)	0.1463 (17)	
H14A	0.6090	−0.1531	0.1020	0.219*	
H14B	0.5337	−0.1076	0.0242	0.219*	
H14C	0.5367	−0.0501	0.1211	0.219*	
N1	0.83129 (15)	0.08188 (15)	−0.02441 (14)	0.0603 (5)	
O1	0.92925 (15)	0.15576 (15)	−0.14407 (11)	0.0756 (5)	
O2	1.08678 (12)	0.16444 (13)	0.15127 (10)	0.0655 (5)	
O3	0.66204 (14)	−0.01773 (15)	0.04411 (16)	0.0967 (7)	

### Atomic displacement parameters (Å^2^)

**Table:**

	*U*^11^	*U*^22^	*U*^33^	*U*^12^	*U*^13^	*U*^23^
C1	0.102 (2)	0.0731 (17)	0.0625 (18)	0.0109 (16)	0.0183 (17)	0.0169 (14)
C2	0.0779 (17)	0.0560 (14)	0.0623 (16)	0.0018 (12)	0.0185 (14)	0.0051 (12)
C3	0.0573 (13)	0.0387 (10)	0.0461 (12)	0.0039 (9)	0.0084 (10)	−0.0004 (9)
C4	0.0533 (13)	0.0420 (11)	0.0475 (13)	0.0008 (9)	0.0042 (11)	−0.0061 (9)
C5	0.0569 (13)	0.0397 (11)	0.0487 (13)	0.0001 (10)	0.0111 (11)	−0.0039 (9)
C6	0.0844 (18)	0.0595 (14)	0.0515 (15)	−0.0051 (13)	0.0160 (13)	−0.0006 (11)
C7	0.109 (2)	0.0672 (17)	0.0693 (18)	−0.0039 (16)	0.0394 (18)	0.0059 (14)
C8	0.076 (2)	0.0610 (16)	0.114 (3)	−0.0144 (14)	0.0458 (18)	−0.0081 (16)
C9	0.0574 (15)	0.0513 (13)	0.097 (2)	−0.0045 (12)	0.0184 (15)	−0.0099 (14)
C10	0.0535 (14)	0.0390 (11)	0.0647 (16)	0.0026 (10)	0.0100 (12)	−0.0055 (10)
C12	0.0681 (16)	0.0475 (12)	0.0476 (14)	0.0119 (11)	−0.0006 (12)	−0.0002 (10)
C13	0.0597 (15)	0.0896 (19)	0.0872 (19)	−0.0186 (14)	0.0017 (14)	−0.0050 (15)
C14	0.097 (2)	0.120 (3)	0.221 (5)	−0.061 (2)	0.023 (3)	−0.039 (3)
N1	0.0608 (13)	0.0559 (12)	0.0641 (14)	0.0049 (9)	−0.0062 (10)	−0.0050 (10)
O1	0.0975 (14)	0.0816 (12)	0.0476 (10)	0.0149 (10)	−0.0040 (10)	0.0084 (8)
O2	0.0644 (10)	0.0786 (11)	0.0535 (10)	−0.0168 (8)	0.0009 (8)	−0.0068 (8)
O3	0.0616 (11)	0.0872 (13)	0.1413 (19)	−0.0202 (10)	0.0027 (12)	−0.0185 (12)

### Geometric parameters (Å, °)

**Table:**

C1—C2	1.316 (3)	C8—C9	1.358 (4)
C1—O1	1.375 (3)	C8—H8	0.9300
C1—H1	0.9300	C9—O3	1.372 (3)
C2—C3	1.446 (3)	C9—C10	1.422 (3)
C2—H2	0.9300	C10—N1	1.364 (3)
C3—C4	1.378 (3)	C12—N1	1.296 (3)
C3—C12	1.404 (3)	C12—O1	1.372 (3)
C4—O2	1.350 (2)	C13—O2	1.429 (3)
C4—C5	1.422 (3)	C13—H13A	0.9600
C5—C6	1.410 (3)	C13—H13B	0.9600
C5—C10	1.418 (3)	C13—H13C	0.9600
C6—C7	1.361 (3)	C14—O3	1.420 (3)
C6—H6	0.9300	C14—H14A	0.9600
C7—C8	1.397 (4)	C14—H14B	0.9600
C7—H7	0.9300	C14—H14C	0.9600
			
C2—C1—O1	113.2 (2)	C8—C9—C10	120.9 (3)
C2—C1—H1	123.4	O3—C9—C10	114.3 (2)
O1—C1—H1	123.4	N1—C10—C5	123.87 (19)
C1—C2—C3	106.5 (2)	N1—C10—C9	118.1 (2)
C1—C2—H2	126.7	C5—C10—C9	118.0 (2)
C3—C2—H2	126.7	N1—C12—O1	119.3 (2)
C4—C3—C12	115.35 (19)	N1—C12—C3	131.0 (2)
C4—C3—C2	139.6 (2)	O1—C12—C3	109.8 (2)
C12—C3—C2	105.1 (2)	O2—C13—H13A	109.5
O2—C4—C3	126.58 (19)	O2—C13—H13B	109.5
O2—C4—C5	114.85 (19)	H13A—C13—H13B	109.5
C3—C4—C5	118.56 (19)	O2—C13—H13C	109.5
C6—C5—C10	119.8 (2)	H13A—C13—H13C	109.5
C6—C5—C4	121.8 (2)	H13B—C13—H13C	109.5
C10—C5—C4	118.35 (19)	O3—C14—H14A	109.5
C7—C6—C5	119.7 (2)	O3—C14—H14B	109.5
C7—C6—H6	120.1	H14A—C14—H14B	109.5
C5—C6—H6	120.1	O3—C14—H14C	109.5
C6—C7—C8	121.3 (3)	H14A—C14—H14C	109.5
C6—C7—H7	119.3	H14B—C14—H14C	109.5
C8—C7—H7	119.3	C12—N1—C10	112.91 (19)
C9—C8—C7	120.2 (2)	C12—O1—C1	105.51 (19)
C9—C8—H8	119.9	C4—O2—C13	119.57 (18)
C7—C8—H8	119.9	C9—O3—C14	116.6 (3)
C8—C9—O3	124.8 (2)		
			
O1—C1—C2—C3	0.2 (3)	C4—C5—C10—C9	179.04 (18)
C1—C2—C3—C4	−178.6 (2)	C8—C9—C10—N1	−179.6 (2)
C1—C2—C3—C12	−0.2 (2)	O3—C9—C10—N1	0.7 (3)
C12—C3—C4—O2	−178.62 (18)	C8—C9—C10—C5	1.4 (3)
C2—C3—C4—O2	−0.3 (4)	O3—C9—C10—C5	−178.24 (18)
C12—C3—C4—C5	0.4 (3)	C4—C3—C12—N1	−0.1 (3)
C2—C3—C4—C5	178.7 (2)	C2—C3—C12—N1	−179.0 (2)
O2—C4—C5—C6	−1.2 (3)	C4—C3—C12—O1	178.92 (17)
C3—C4—C5—C6	179.67 (19)	C2—C3—C12—O1	0.1 (2)
O2—C4—C5—C10	178.73 (17)	O1—C12—N1—C10	−179.06 (18)
C3—C4—C5—C10	−0.4 (3)	C3—C12—N1—C10	−0.1 (3)
C10—C5—C6—C7	0.1 (3)	C5—C10—N1—C12	0.1 (3)
C4—C5—C6—C7	−179.9 (2)	C9—C10—N1—C12	−178.82 (18)
C5—C6—C7—C8	0.4 (4)	N1—C12—O1—C1	179.27 (19)
C6—C7—C8—C9	0.0 (4)	C3—C12—O1—C1	0.1 (2)
C7—C8—C9—O3	178.7 (2)	C2—C1—O1—C12	−0.2 (3)
C7—C8—C9—C10	−1.0 (4)	C3—C4—O2—C13	−1.7 (3)
C6—C5—C10—N1	−179.89 (19)	C5—C4—O2—C13	179.32 (19)
C4—C5—C10—N1	0.2 (3)	C8—C9—O3—C14	−10.6 (4)
C6—C5—C10—C9	−1.0 (3)	C10—C9—O3—C14	169.1 (2)
